# *Drosophila melanogaster* as a Model for Gastrointestinal Radiation Injury: Conserved Mechanisms, Experimental Approaches, and Countermeasure Discovery

**DOI:** 10.3390/ijms27177949

**Published:** 2026-09-07

**Authors:** Robert P. Volpe, Tomoko Y. Steen

**Affiliations:** School of Medicine, Georgetown University, Washington, DC 20057, USA

**Keywords:** *Drosophila melanogaster*, gastrointestinal acute radiation syndrome, intestinal stem cells, epithelial barrier, microbiome, ionizing radiation, radiation countermeasures

## Abstract

The gastrointestinal tract is a critical target of acute radiation exposure. Severe radiation exposure can deplete epithelial stem and progenitor cells, compromise barrier integrity, alter host–microbe interactions, and drive fluid loss, inflammation, and systemic decline. Mammalian models remain essential for clinical translation, but their cost and complexity constrain sample sizes, statistical power, large-scale mechanistic discovery, and countermeasure screening. The adult *Drosophila melanogaster* midgut provides a complementary in vivo platform containing intestinal stem cells, absorptive enterocytes, enteroendocrine cells, epithelial junctions, an associated microbiota, and conserved innate immune and injury-response pathways. Direct irradiation studies have demonstrated DNA damage, altered stem cell proliferation and differentiation, epithelial plasticity, apoptosis, autophagy-associated responses, morphological disruption, barrier failure, microbiome changes, and reduced survival. These phenotypes can be modulated by genotype, sex, diet, microbial status, antioxidant capacity, and regenerative signaling. This review evaluates the biological rationale, direct evidence, experimental assays, and countermeasure applications supporting the fly midgut as a model for studying gastrointestinal radiation injury. Although *Drosophila* has a long history of use in radiation research, our analysis indicates that the fly midgut is best positioned not as a miniature model of clinical gastrointestinal acute radiation syndrome, but as a genetically precise and scalable system for identifying conserved mechanisms and prioritizing interventions for validation in mammalian models.

## 1. Introduction

The gastrointestinal (GI) tract forms a dynamic interface between the body and the external environment, integrating digestion, nutrient absorption, barrier defense, immune regulation, and tissue renewal. Its healthy functioning depends on continuous epithelial replacement from stem and progenitor cells as well as a regulated relationship with the microbial communities inhabiting the lumen. These same properties make the gut acutely vulnerable to ionizing radiation. When injury overwhelms epithelial renewal, the intestine can no longer maintain the physical and immunological boundary separating the host from the luminal environment. The resulting gastrointestinal acute radiation syndrome (GI-ARS) is a severe and often fatal condition marked by nausea, diarrhea, dehydration, electrolyte imbalance, bacterial translocation, sepsis, and systemic decline [1,2,3,4,5,6,7,8].

The classical view of GI-ARS centers on depletion of rapidly proliferating intestinal crypt stem and progenitor cells. This remains central to the syndrome, but gastrointestinal radiation injury is not merely a problem of epithelial cell loss. It is a failure of tissue homeostasis arising from DNA damage, oxidative stress, impaired regeneration, altered differentiation, breakdown of epithelial junctions, innate immune activation, microbiome disruption, and metabolic deterioration. These processes are interdependent. Epithelial damage weakens the barrier; barrier failure permits microbial products and organisms to enter host tissues; microbial translocation and dysbiosis amplify inflammation; and inflammation further compromises repair. Models of gastrointestinal radiation injury must therefore capture not only cell death, but also the dynamic relationship among epithelial renewal, barrier integrity, host defense, and the microbiome [7,8].

Mammalian models have been indispensable for defining the pathophysiology and management of radiation injury. Rodents, minipigs, and non-human primates remain essential for translational validation, dose–response modeling, medical management studies, and regulatory development of radiation countermeasures [2,4,5,6]. At the same time, these systems impose substantial financial, ethical, logistical, and statistical constraints. They are poorly suited for large-scale genetic interrogation or rapid screening of dietary, microbial, pharmacologic, and environmental modifiers. Their anatomical and immunological complexity can also make it difficult to determine which components of injury are primary and which arise secondarily from systemic collapse.

*Drosophila melanogaster* may not appear to be an intuitive model for studying gastrointestinal radiation injury. Flies differ from humans at both tissue and organismal levels, lacking crypt–villus architecture and adaptive immunity while also differing in size, lifespan, anatomy, fluid physiology, and microbial ecology. Nevertheless, *Drosophila* has a long and productive history in radiation biology, beginning with foundational studies of radiation-induced mutagenesis and extending into modern work on DNA damage responses, oxidative stress, tissue degeneration, aging, and spaceflight biology. More importantly, the adult fly midgut preserves many of the biological functions that determine whether an irradiated gastrointestinal epithelium fails or recovers. Adult *Drosophila* can tolerate ionizing-radiation doses far above those that are lethal to mammals, making biological responses and mechanisms more informative for cross-species comparison than direct numerical dose equivalence. The present review focuses specifically on the gastrointestinal epithelium, direct intestinal phenotypes, and countermeasure discovery. The *Drosophila* midgut is a self-renewing epithelial organ composed principally of absorptive enterocytes, hormone-producing enteroendocrine cells, enteroblast progenitors, and proliferative intestinal stem cells (ISCs). These cells occupy a regionally specialized digestive tract that performs nutrient absorption, endocrine signaling, innate immune surveillance, barrier maintenance, and host–microbe regulation. Several pathways governing epithelial renewal and injury response in the fly gut have functional counterparts in mammalian intestinal homeostasis and repair. These include JAK/STAT [9,10,11,12], stress-activated JNK and p38 MAPK signaling [13,14,15], EGFR/Ras/MAPK [16,17,18,19], Wnt/Wingless [20,21,22,23,24], Notch [25,26,27,28,29], and Hippo/Yorkie [30,31,32,33,34]. The fly midgut is anatomically simpler than the mammalian intestine, but this simplicity can be experimentally useful because epithelial injury, stem cell behavior, barrier failure, and microbial modulation can be interrogated directly in an intact animal.

Large, statistically robust cohorts of flies can be irradiated, maintained, genetically manipulated, and phenotyped with relative ease. Radiation responses can be measured across organismal, tissue, cellular, and molecular scales, including survival, lifespan, feeding behavior, gut morphology, ISC proliferation, epithelial dysplasia, DNA damage signaling, oxidative stress, antimicrobial peptide induction, microbiome composition, and barrier integrity. Germ-free, antibiotic-treated, gnotobiotic, and diet-controlled flies further allow investigators to test how defined microbial states influence radiation susceptibility and recovery [35,36].

The value of *Drosophila* is therefore not that it reproduces mammalian GI-ARS in miniature. Its value lies in the ability to isolate and manipulate conserved components of radiation-induced gut failure. The model permits focused interrogations that are difficult to approach at scale in vertebrates: Which epithelial pathways determine survival after irradiation? How do ISCs balance arrest, repair, proliferation, differentiation, and apoptosis after DNA damage? How does barrier failure contribute to systemic decline? Which microbial communities or metabolites worsen or mitigate injury? Which dietary or pharmacologic interventions preserve gut structure and function after exposure?

This review evaluates where the adult *Drosophila melanogaster* midgut is most useful for studying gastrointestinal radiation injury and where its translational limits lie. We first consider its historical foundation in radiation research and then examine the adult midgut as a model gastrointestinal epithelium. We next synthesize direct evidence for radiation-induced intestinal injury, evaluate experimental approaches for measuring damage and recovery, and consider applications in countermeasure discovery. Finally, we define the limitations that must guide translation and identify priorities for future work. The central argument is that *Drosophila* should be used neither as a replacement for vertebrate models nor as an anatomical facsimile of the mammalian intestine, but as a genetically precise and scalable discovery platform whose most promising findings can be advanced into organoid, rodent, and large-animal systems.

This article is a narrative review rather than a systematic review. The literature was identified through targeted searches of PubMed using combinations of terms including *Drosophila melanogaster*, radiation, irradiation, ionizing radiation, intestine, intestinal, gut, and midgut, supplemented by examination of relevant references cited within identified studies. The literature available through 1 July 2026 was considered. Studies were prioritized when they directly examined ionizing-radiation effects on the *Drosophila* gastrointestinal system or provided mechanistically relevant evidence concerning intestinal injury, repair, barrier function, microbiome interactions, biological modifiers, or candidate countermeasures; incidental keyword matches and studies unrelated to ionizing radiation were excluded. Two studies included in the evidence synthesis [37,38] were authored by R.P.V.; these studies were included according to the same relevance criteria applied to the other literature.

## 2. Drosophila in Radiation Biology: From Mutagenesis to Tissue Injury

*Drosophila* was among the first animals used to establish the biological consequences of ionizing radiation. In 1927 and 1928, Hermann J. Muller used X-ray-irradiated flies to demonstrate radiation-induced mutation and quantify the relationship between radiation exposure and mutation frequency [39,40]. These experiments helped move radiation biology from descriptive observation toward quantitative mechanistic analysis. During the early U.S. V-2 research program, fruit flies were carried to high altitude in experiments investigating the biological effects of radiation above the atmosphere [41]. The model has since remained useful whenever radiobiology has required large cohorts, genetic resolution, or separation of complex phenotypes into experimentally tractable components.

For much of its history, fly radiobiology emphasized mutagenesis, development, fertility, and survival. More recent studies have extended this work to persistent organismal and tissue-level phenotypes. Developmental irradiation can produce dose-dependent reductions in adult survival, locomotor impairment, and apoptosis, demonstrating that radiation injury can be followed from an initiating exposure throughlater functional decline [42]. Contemporary work in the intestine similarly moves beyond mutation frequency and lethality to examine DNA-damage signaling, epithelial renewal, barrier function, immune activation, microbiome state, and cell-specific injury responses.

The earliest fly studies established that radiation effects could be quantified and genetically interrogated. Modern intestinal studies apply the same experimental logic to a multicellular organ: a phenotype is defined, natural or engineered variation is introduced, and the mechanisms controlling injury or recovery are resolved. The adult midgut therefore represents not a departure from the historical strengths of *Drosophila* radiobiology, but an extension of them from the gene and organism to tissue homeostasis.

## 3. The Adult Drosophila Midgut as a Model Gastrointestinal Epithelium

The adult *Drosophila* midgut is the portion of the fly digestive tract most relevant to gastrointestinal radiation injury. Its modeling value does not depend on structural equivalence to the mammalian small intestine or colon. Rather, it preserves properties central to the determination of intestinal response to injury: a renewing epithelium, resident stem cells, regionally specialized digestive and absorptive compartments, epithelial junctions that regulate permeability, innate immune surveillance, and continuous interaction with diet and microbiota. These features allow radiation injury to be studied as a failure of epithelial maintenance, barrier defense, and regenerative recovery in an intact animal.

### 3.1. Regional and Cellular Organization

**Figure 1:**
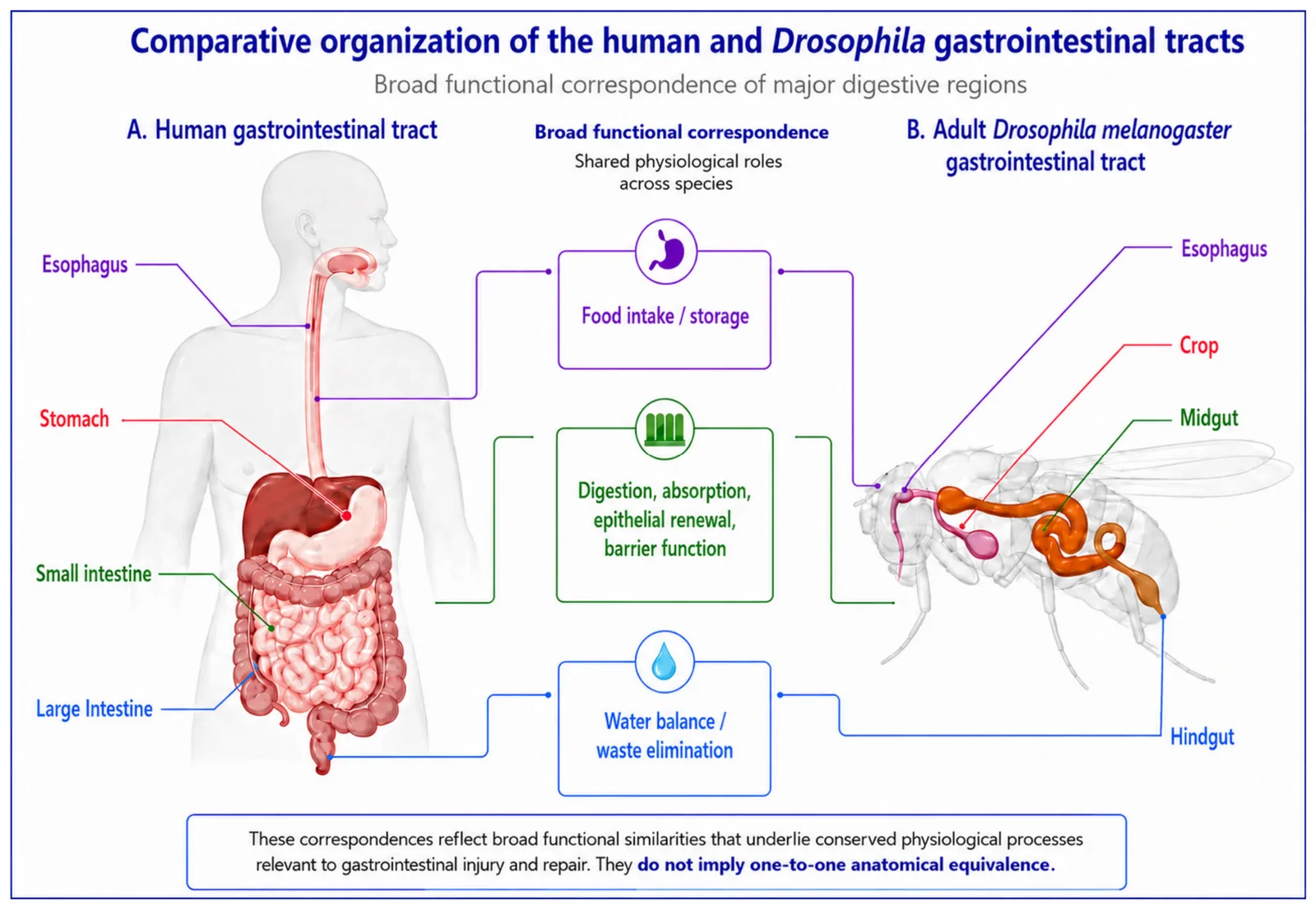
Comparative organization of the human and adult *Drosophila melanogaster* gastrointestinal tracts. Broad functional correspondences include food intake and storage; digestion, absorption, epithelial renewal, and barrier function; and water balance and waste elimination. These relationships indicate conserved physiological roles rather than one-to-one anatomical equivalence.

The adult fly digestive tract is divided into foregut, midgut, and hindgut compartments, with the midgut serving as the principal site of digestion and nutrient absorption. Detailed morphologic and transcriptomic analyses have shown that the midgut is highly regionalized. Major anterior, middle, and posterior domains, further divided into functionally distinct subregions, differ in morphology, pH, digestive enzyme expression, nutrient transport, immune tone, and stem cell behavior [43,44]. This organization matters for radiation studies because intestinal injury may not be uniform along the gut. A treatment that preserves one region may fail in another, and measurements restricted to the posterior midgut may not describe the entire organ. Broad functional correspondences between major digestive regions in humans and adult *Drosophila* are illustrated in Figure 1.

The adult midgut epithelium is maintained by ISCs positioned along its basal surface. ISC division generates self-renewing stem cells and progeny that replenish differentiated epithelial populations. In the canonical lineage, an enteroblast differentiates into an absorptive enterocyte, whereas enteroendocrine cells arise through a related lineage choice regulated in substantial part by Delta-Notch signaling [25,26]. Enterocytes make up most of the epithelial surface and perform digestion, absorption, xenobiotic handling, and barrier-associated functions. Enteroendocrine cells are less abundant but provide peptide and hormonal signals that regulate local physiology and systemic metabolism. For radiation biology, this organization permits injury to be mapped to distinct fates: stem cell loss or arrest, progenitor expansion, failed differentiation, enterocyte damage, endocrine disruption, cellular reprogramming, or dysplastic repair.

A central strength of the model is that routine epithelial renewal can be distinguished from the regenerative response triggered by injury. Under basal conditions, epithelial turnover is coordinated with feeding, metabolism, and microbial exposure. After damage, stressed or dying enterocytes and neighboring cells release signals that stimulate ISC proliferation and epithelial replacement. The tissue must remove damaged cells, preserve enough continuity to maintain the barrier, and replace lost cells without producing disorganized growth. Radiation can disrupt any of these steps.

### 3.2. Regenerative Niche and Conserved Injury-Response Signaling

The ISC compartment is regulated by a distributed niche rather than a single anatomically discrete structure. Enterocytes, enteroblasts, enteroendocrine cells, and visceral muscle all provide signals that influence stem cell proliferation, regional identity, differentiation, and survival. This multicellular architecture is useful for GI radiobiology because an apparent stem cell phenotype may arise through direct ISC damage or through altered signals from differentiated cells and surrounding tissues. An intervention that improves recovery may protect stem cells, reduce enterocyte stress, preserve junctional organization, modify microbial signaling, or restore niche-derived growth signals. Cell-specific genetic tools allow these possibilities to be separated.

The translational value of the fly midgut is strongest when it is framed at the level of conserved injury-response modules rather than one-to-one anatomical equivalence. Ionizing radiation challenges both fly and mammalian intestinal epithelia to detect genotoxic and oxidative stress, control inflammatory tone, preserve barrier selectivity, coordinate host–microbe signaling, and regenerate lost or dysfunctional cells. Several conserved signaling pathways provide experimentally accessible modules for studying epithelial renewal and repair. These include JAK/STAT [9,10,11,12], stress-activated JNK and p38 MAPK signaling [13,14,15], EGFR/Ras/ERK [16,17,18,19], Wnt/Wingless [20,21,22,23,24], Notch [25,26,27,28,29], and Hippo/Yorkie [30,31,32,33,34]. Radiation-relevant responses can also be examined through innate immune signaling [35,45], antioxidant defenses [14,35,37,38], and DNA-damage and cell-fate pathways [45,46,47,48,49].

These pathways should not be viewed as independent modules. In the injured Drosophila intestine, stress-responsive JNK signaling can promote expression of Upd-family cytokines, which in turn activate JAK/STAT signaling in ISCs and stimulate regenerative proliferation; EGFR/Ras/ERK and Hippo/Yorkie signaling can further converge on epithelial growth and repair responses [9,13,16,17,30,31,32]. The extent and direction of this pathway crosstalk are context dependent, however, and individual signaling components need not respond uniformly to irradiation.

Conservation does not imply that pathway output will be identical across species or injury contexts. A pathway that promotes repair at one dose or time point may contribute to apoptosis, hyperplasia, or dysplasia under another. For this reason, pathway activity should be interpreted together with tissue function. Increased ISC proliferation, for example, may reflect successful regeneration, compensation for ongoing cell loss, or disordered growth by damaged progenitors. The fly model is valuable precisely because pathway activation can be paired with lineage, morphology, permeability, and survival endpoints. Representative pathways, readouts, and translational interpretations are summarized in Table 1.

### 3.3. Barrier Function, Innate Immunity, and Host–Microbe Interactions

Barrier biology provides another major point of relevance. Mammalian epithelial barrier function depends on tight and adherens junctions, mucus, immune effectors, vascular and stromal support, and regional epithelial specialization. In *Drosophila*, smooth septate junctions provide the principal paracellular seal, while the peritrophic matrix forms an acellular chitin-rich layer between luminal contents and the epithelial surface. Septate-junction proteins such as Snakeskin and Mesh contribute both to barrier integrity and to signaling that regulates epithelial homeostasis [50]. These structures are not directly equivalent to mammalian tight junctions or mucus, but they serve a comparable physiological functions in maintaining a selective barrier between host tissue and the gut lumen.

The fly gut also lacks adaptive immunity, organized lymphoid structures, Paneth cells, and the full cellular complexity of mammalian mucosal immunity. Nevertheless, it mounts robust epithelial innate immune responses through Imd/Relish and Toll/Dif signaling, antimicrobial peptide production, reactive oxygen species, and microbiome-sensitive stress pathways. These systems support mechanistic analysis of how epithelial damage, microbial exposure, and inflammatory signaling interact after irradiation.

The *Drosophila* microbiota is less complex and often less stable than that of mammals. This limits direct ecological translation but creates substantial experimental leverage. Microbial communities can be depleted, restored, or defined, and diet can be controlled in parallel. Axenic and gnotobiotic animals therefore permit causal tests of whether radiation phenotypes depend on microbial presence, individual taxa, community composition, or microbe-derived metabolites [35,36]. The adult midgut occupies a useful middle ground between reductionist epithelial culture systems and complex vertebrate models: it retains feeding, metabolism, microbial exposure, tissue renewal, barrier function, and organismal survival while remaining genetically tractable and scalable.

## 4. Direct Evidence of Radiation-Induced Intestinal Injury in Drosophila

Although *Drosophila melanogaster* has been used in radiation research for nearly a century, the literature directly examining radiation injury in the adult intestine remains comparatively small. Nevertheless, the available evidence is sufficient to establish that the fly midgut responds to ionizing radiation through measurable changes in DNA-damage signaling, intestinal stem cell activity, epithelial differentiation, cellular morphology, barrier function, oxidative stress, immune activation, host–microbe interactions, and organismal survival. The studies that define these responses differ substantially in radiation source, dose, dose rate, developmental stage, genotype, sex, microbial status, and time of analysis. They should therefore not be treated as points along a single, continuous dose–response curve. A dose that produces a particular phenotype in one fly protocol is not directly equivalent to the same dose in another experimental system, and it should not be interpreted as a human-equivalent exposure. The more useful question is whether irradiation produces a reproducible and mechanistically interpretable phenotype that can be genetically, environmentally, or pharmacologically modified.

Radiation does not produce a single uniform state of intestinal failure in Drosophila. Instead, the midgut can enter several overlapping injury-response states, including compensatory proliferation, proliferative arrest, cell death, abnormal differentiation, cellular plasticity, inflammatory signaling, and persistent barrier dysfunction. Which state predominates depends on the severity of the exposure, the time at which the intestine is examined, and the biological condition of the animal. This variability is not merely experimental noise. It reflects the central biological problem being modeled: following irradiation, the intestinal epithelium must determine which cells can be repaired, which cells must be removed, whether surviving progenitors should divide, and whether epithelial replacement can occur without compromising tissue organization and barrier function.

### 4.1. DNA Damage and Intestinal Stem Cell Responses

The proliferative behavior of intestinal stem cells after irradiation is strongly dependent on dose and experimental context. Pyo and colleagues directly examined adult midgut ISCs following ionizing radiation and found that 10 Gy reduced their proliferative capacity. In contrast, exposure to 2 Gy increased ISC proliferation while also increasing γH2Av and 8-oxo-deoxyguanosine, markers of DNA damage and oxidative injury. The lower exposure was further associated with increased JNK and AKT signaling, abnormal differentiation, and centrosome amplification in ISCs. Thus, increased proliferation following radiation did not necessarily represent successful regeneration. It occurred together with persistent molecular injury and abnormalities that could compromise the fidelity of epithelial renewal [46].

Regeneration is often evaluated by asking whether stem cells divide after injury. However, proliferation by itself is not an adequate measure of recovery. An irradiated ISC may divide while carrying unrepaired DNA damage, producing abnormal daughter cells, or entering a state of dysregulated growth. Conversely, a reduction in mitotic activity may represent reversible checkpoint activation, loss of the stem cell population, or an inability of surviving cells to respond to regenerative signals. The direction of the proliferative response therefore cannot be interpreted without simultaneous measurements of DNA damage, cell death, differentiation, and tissue function.

At a higher exposure, Sharma and colleagues observed a different pattern. Adult flies exposed to 100 Gy showed rapid accumulation of γH2Av foci in the intestine, followed by apoptosis in multiple epithelial populations. Delta-positive ISCs underwent apoptosis one day after irradiation, but cell death was not restricted to the stem cell compartment. Enterocytes and enteroendocrine cells also displayed evidence of apoptosis, and the pro-apoptotic genes *hid* and *reaper* were increased in dissected intestines. Mitotic activity, measured by phospho-histone H3 staining, was reduced as early as one day after irradiation and remained impaired at 14 days. The abundance of Delta-positive ISCs was likewise reduced at both time points. In this setting, radiation did not merely damage differentiated epithelial cells and provoke a compensatory response. It depleted or disabled part of the regenerative compartment itself [45].

Other work indicates that stem cells are not uniformly susceptible to radiation-induced apoptosis. Xing and colleagues found that adult stem cells in the Drosophila midgut and germline could be relatively resistant to radiation-induced apoptosis compared with surrounding differentiated cells. Their mechanistic experiments identified signaling from dying daughter cells through a Pvf1-Tie-bantam-Hid survival axis, although the detailed pathway analysis was performed primarily in the germline. The extent to which the same signaling mechanism protects irradiated midgut ISCs remains to be established directly. Nevertheless, the study demonstrates that radiation susceptibility can differ between stem cells and their differentiated progeny, and that dying cells may actively influence the survival state of neighboring regenerative cells rather than serving only as passive evidence of tissue damage [47].

DNA-damage-response studies using irradiation as a defined experimental challenge provide a complementary view of regenerative competence. Park and colleagues exposed adult flies to 5 Gy of gamma radiation and detected increased pS/TQ and γH2AvD signals in ISCs and enteroblasts. Cell-specific depletion of ATM or ATR indicated that both kinases contribute to ISC maintenance and proliferation, with ATR loss producing the stronger phenotype [52].

A related study examined the differentiated epithelial compartment. Irradiation induced γH2AvD in enterocytes as well as progenitor populations, while chronic enterocyte-specific depletion of Mre11, Rad50, Nbs1, ATM, ATR, Chk1, or Chk2 promoted enterocyte death and non-autonomous ISC hyperproliferation, DNA-damage accumulation, and centrosome amplification [53]. These experiments were designed primarily to interrogate DNA-damage-response function rather than to follow intestinal recovery after irradiation, and most of the later phenotypes arose from sustained genetic depletion. Their relevance to radiation injury is therefore mechanistic rather than a direct demonstration of a longitudinal injury course.

These findings should not be read as contradictory. Together, they show that the ISC response is determined by exposure severity, cell state, time after irradiation, intrinsic DNA-damage competence, and signals received from the surrounding epithelium. At one end of the response spectrum, radiation may provoke proliferation that is increased but disordered. At the other, it may produce apoptosis, stem cell depletion, and persistent loss of proliferative competence. Between these states, surviving ISCs may enter temporary arrest or receive protective signals from damaged neighboring cells, while impaired DNA-damage handling in enterocytes can drive compensatory proliferation from otherwise intact progenitors. The relevant endpoint is therefore not simply the number of mitotic cells. It is whether the surviving regenerative compartment can produce an appropriately differentiated and functionally organized epithelium.

### 4.2. Altered Differentiation and Epithelial Plasticity

Radiation injury to the fly intestine is not limited to cell death and stem cell depletion. It can also alter the identity and developmental behavior of surviving epithelial cells. Pyo and colleagues observed that the increased ISC proliferation induced by 2 Gy was accompanied by abnormal differentiation. This finding suggests that radiation can disrupt the coordination between cell division and lineage allocation, producing new epithelial cells without necessarily restoring the correct balance of absorptive and secretory cell types. In this context, an increase in epithelial cellularity could coexist with declining tissue quality [46].

More recent work has extended this concept beyond the conventional ISC–enteroblast lineage. Qian and colleagues showed that ionizing radiation induces cellular plasticity in adult midgut enteroendocrine cells. Radiation activated stress-responsive programs within the enteroendocrine lineage, including expression of the transcription factor Xrp1 and the cytokine gene upd3. Both Xrp1 and upd3 were required for radiation-induced enteroendocrine cell fate conversion, while enteroendocrine-specific Xrp1 expression was sufficient to induce ectopic expression of progenitor-associated genes under otherwise basal conditions. These results indicate that differentiated enteroendocrine cells are not inert casualties of irradiation. They can sense damage, activate regenerative cytokine signaling, dedifferentiate toward a stem-like state, and subsequently redifferentiate toward the enterocyte lineage [48].

These findings expand the conceptual model of gastrointestinal radiation injury in the fly beyond stem cell loss and dysfunction. A stem cell-centered model asks whether irradiation removes or disables the cells responsible for epithelial replacement. A plasticity-centered model adds another possibility: radiation may alter the identity of differentiated cells and recruit them into the injury response. Such plasticity could be adaptive if it expands the number of cells capable of contributing to repair. It could also be maladaptive if the process produces incomplete reprogramming, inappropriate lineage mixtures, or persistent epithelial disorganization.

The existence of radiation-induced plasticity therefore raises questions that cannot be answered by counting cell types at a single time point. It will be important to determine whether converted cells remain viable, whether they generate mature and functional progeny, whether their descendants integrate into the epithelium, and whether this process improves or worsens barrier recovery. The genetic lineage-tracing tools available in Drosophila make these questions experimentally approachable. In this respect, the fly model offers more than a means of quantifying radiation-induced cell loss. It permits direct investigation of how epithelial identity is dismantled, reassigned, and potentially restored after injury.

### 4.3. Epithelial Morphology and Barrier Dysfunction

The functional consequence of gastrointestinal radiation injury is not determined solely by how many epithelial cells remain. The surviving cells must preserve a continuous and selective boundary between the animal and the contents of the gut lumen. Barrier failure therefore provides an important bridge between molecular or cellular injury and organismal decline.

Sharma and colleagues found that adult flies exposed to 100 Gy developed shorter intestines and increased intestinal permeability 14 days after irradiation. Permeability, measured using the Smurf dye-leakage assay, increased with radiation dose and was observed in both males and females. A staggered exposure consisting of four 25 Gy doses administered every other day produced a degree of permeability similar to that observed following a single 100 Gy exposure when assessed at the same post-exposure endpoint. Increased permeability was accompanied by sustained induction of antimicrobial and inflammatory-response genes in the intestine and fat body, linking local epithelial failure to a broader organismal response [45].

These findings establish barrier dysfunction as more than a histological inference. Dye leakage provides a direct functional indication that luminal contents are no longer being confined to the digestive tract. In the fly, as in mammals, this transition is biologically consequential. Once the epithelial barrier becomes permissive, microbes and microbial products can interact with tissues that are normally shielded from them, potentially amplifying immune activation, metabolic disruption, and mortality. The anatomical structures that maintain the barrier differ between flies and mammals, but the physiological question remains comparable: does the irradiated intestine continue to separate the host from its luminal environment?

Subsequent work used the same permeability phenotype to investigate meltrin, a *Drosophila* ortholog of mammalian ADAM19 identified through the DGRP screening framework. Enterocyte-specific meltrin knockdown reduced Smurf positivity after 100 Gy and was accompanied by lower γH2Av-associated DNA-damage signal, β-galactosidase, and upd3 readouts in the irradiated gut. Feeding the broad-spectrum metalloprotease inhibitor batimastat (BB-94) likewise reduced permeability [54].

These findings are mechanistically suggestive but do not demonstrate comprehensive protection. Meltrin knockdown did not improve mean or median lifespan, BB-94 is not specific for ADAM19, and the selected marker panel does not by itself establish canonical mammalian cellular senescence in the fly. Nevertheless, the study demonstrates that a permeability phenotype can be carried from population genetics to intestine-restricted validation and pharmacologic testing [54].

Radiation-induced morphological injury has also been observed directly in enterocytes. Following acute exposure to 1000 Gy, male midguts displayed increased abnormal nuclear morphology, loss of clearly defined actin-associated cellular boundaries, and visible holes within the epithelial actin layer. Females exposed under the same conditions showed fewer nuclear abnormalities and fewer actin-associated holes, although they also developed significant disruption of cellular boundaries. These tissue-level differences corresponded with the greater post-irradiation mortality observed in males, suggesting that sex-specific survival was associated with sex-specific intestinal injury in this protocol [38].

The relationship between individual morphological features and survival, however, was not perfectly uniform. Prefeeding males with *Aureobasidium pullulans* improved post-irradiation survival and reduced the frequency of abnormal enterocyte nuclei. The same treatment did not completely preserve actin-defined cellular boundaries, and fungal feeding in the absence of irradiation produced a mixed morphological phenotype. These results indicate that different components of epithelial organization can respond independently. Nuclear morphology, cellular shape, junction-associated organization, tissue continuity, and whole-gut permeability may be related, but they are not interchangeable readouts [38].

This distinction should guide interpretation of future studies. A treatment that reduces abnormal nuclei may not restore barrier function. A treatment that delays dye leakage may not eliminate molecular damage. Conversely, a gut that appears disorganized by microscopy may retain sufficient functional continuity to support survival. The most informative studies will therefore combine morphological analysis with a direct permeability assay and an organismal endpoint. Barrier failure is central to gastrointestinal radiation injury, but no single image or dye assay can fully describe the process by which the barrier deteriorates.

### 4.4. Oxidative Stress, Apoptosis, Autophagy, and Immune Signaling

Radiation-induced intestinal injury is initiated by molecular damage but unfolds through an interconnected stress-response network. DNA lesions, oxidative injury, apoptosis, autophagy, regenerative cytokine signaling, innate immunity, and microbial activity can occur in parallel and influence one another. The fly model is particularly useful for separating these processes because they can be measured in defined cell types and manipulated genetically in the intact animal.

Following 100 Gy irradiation, Sharma and colleagues observed γH2Av accumulation, increased expression of hid and reaper, apoptosis in both progenitor and differentiated epithelial cells, and delayed induction of the damage-responsive cytokine upd3. Radiation also increased intestinal Diptericin expression and fat-body expression of Drosomycin, indicating activation of local and systemic innate immune programs. Not every canonical stress pathway responded in the same manner. Expression of puckered, commonly used as a readout associated with JNK activity, was not increased in that experimental setting. This negative result demonstrates that pathways activated by infection, aging, or chemical damage cannot be assumed to respond identically following irradiation [45].

Autophagy represents another component of the intestinal radiation response. Trinca and Malik exposed young adult flies to 150 Gy of gamma radiation and examined enterocyte expression of an mCherry-tagged Atg8 reporter one day later. Irradiated midguts showed increased Atg8 signal throughout much of the organ, with multiple cytoplasmic puncta present in enterocytes. Whole-tissue reporter intensity was approximately twofold higher than in nonirradiated controls. These findings demonstrate that irradiation alters an autophagy-associated marker in the adult midgut [49].

The interpretation of this response requires caution. Increased Atg8 abundance or puncta formation is consistent with increased autophagosome formation, but it does not by itself establish increased autophagic flux. The same pattern could arise if autophagosomes are produced more rapidly, degraded more slowly, or both. It also does not establish whether autophagy is protective or contributes to tissue loss. Resolving these possibilities will require paired measurements of autophagosome formation and lysosomal turnover, together with cell-specific genetic manipulation of the autophagy pathway. The result identifies a tractable cellular response that occurs before overt lethality and can be experimentally tested for causal importance.

Oxidative stress is likewise closely connected to microbial status. Lee and colleagues irradiated third-instar larvae with 0.1 or 5 Gy and later examined adult microbial composition, physiology, and intestinal oxidative responses. Irradiation altered bacterial abundance and reduced measures of microbial diversity. Conventional and axenic animals did not respond identically. Although irradiation increased intestinal reactive oxygen species in both groups, the later oxidative and mitochondrial responses were greater in axenic flies. Mean lifespan declined with increasing dose in axenic animals but not in conventional flies under the conditions tested, leading the authors to propose that commensal microbes provided a degree of radioprotection in this experimental context [35].

Zhang and colleagues more directly tested a defined bacterial intervention in adult flies. Females fed *Lactobacillus reuteri* before and after 100 Gy irradiation showed improved survival together with attenuation of several intestinal phenotypes, including barrier failure, epithelial reactive oxygen species, oxidative stress measures, and altered ISC abundance. The intervention also modified AMPK/mTOR- and autophagy-associated gene expression [55].

The design supports an association between a defined probiotic preparation and improved intestinal recovery, but it does not cleanly distinguish radioprotection from mitigation because feeding began before exposure and continued afterward. The study also compared preparations with nonidentical effects, and intestine-restricted causal tests were not performed. The active component and dominant mechanism therefore remain unresolved.

Together, these studies complicate any simple model in which microbes become uniformly harmful after irradiation. Barrier failure may permit microbial translocation and inflammatory amplification, but commensal or probiotic organisms may also contribute metabolites, tonic signals, or redox-regulatory effects that support recovery. The impact of the microbiome is therefore likely to depend on community composition, developmental stage at exposure, intervention timing, diet, barrier integrity, and the time after irradiation. Microbial depletion, microbial overgrowth, and microbial supplementation may each be detrimental or beneficial through different mechanisms [35,55].

Additional evidence supports a role for oxidative defense in organismal radiation resistance. Catalase-null flies showed reduced survival following acute irradiation, consistent with the importance of peroxide detoxification. Dietary manganese increased survival in irradiated males without increasing MnSOD2 protein abundance, suggesting that redox protection can also arise from nonenzymatic manganese-containing metabolite complexes rather than simply from increased antioxidant enzyme expression [37,38]. These findings do not establish that all of the survival effect originates in the intestine, but they identify redox state as a biologically modifiable component of the whole-animal response.

Taken together, the evidence shows that the irradiated midgut does not progress through a simple sequence of DNA damage followed by cell death. It enters a dynamic state in which damaged cells may die, arrest, activate cytokine signaling, engage autophagy, alter immune tone, or interact differently with the resident microbiota. The outcome depends on whether these responses restore epithelial organization or instead amplify tissue dysfunction.

### 4.5. Organismal Outcomes and Biological Modifiers

The most distinctive advantage of Drosophila is not merely that radiation injury can be observed, but that the factors controlling that injury can be discovered at scale. Genetic background, sex, diet, microbial status, antioxidant capacity, and regenerative signaling can all modify post-irradiation outcomes. These variables are often treated as confounders. In the fly, they can instead become experimental entry points.

Sharma and colleagues used 156 strains from the Drosophila Genetic Reference Panel to examine natural variation in radiation-induced intestinal permeability. The strains differed widely in the proportion of animals developing dye leakage after irradiation. This screen identified musashi (msi) as a candidate regulator of the response. ISC-specific reduction of msi worsened permeability and reduced survival, while increased msi expression stimulated ISC proliferation and reduced radiation-associated barrier dysfunction. Increasing ISC proliferation through Cyclin E expression also improved permeability, supporting a causal connection between regenerative competence and maintenance of the intestinal barrier in this model [45].

Bar and colleagues extended this discovery logic with a second DGRP-derived candidate, meltrin. Enterocyte-restricted knockdown and pharmacologic inhibition reduced radiation-associated permeability and selected damage- and senescence-associated readouts, although lifespan was unchanged [54]. The contrast is informative: a modifier can preserve a gut-specific endpoint without overcoming the total organismal burden of exposure.

These studies illustrate the discovery function of the fly particularly well. The entry phenotype was not mutation frequency or nonspecific lethality. It was physiologically relevant intestinal permeability measured across a genetically diverse population, followed by cell-specific validation of candidate mechanisms. Musashi and meltrin then define different translational routes: one centers on regenerative competence, while the other nominates a conserved metalloprotease axis that can be tested pharmacologically. The fly does not complete that translational process, but it can greatly narrow the set of mechanisms and targets advanced for mammalian validation.

Sex is another important modifier, although its effects are not identical across protocols. Sharma and colleagues found that the effects of 100 Gy on survival and permeability occurred in both sexes without a strong sex-specific difference. In contrast, studies using 700 to 1000 Gy gamma radiation found that males were substantially more sensitive than females. Male susceptibility was associated with greater intestinal nuclear and actin-associated abnormalities, lower abundance of high-symmetry manganese metabolite complexes, and a larger survival benefit from dietary manganese or *A. pullulans* [37,38].

These findings should not be forced into a single conclusion that one sex is intrinsically more radiosensitive under all conditions. Rather, they indicate that sex can interact with dose, genetic background, age, diet, endpoint, and post-exposure interval. Sex differences may become visible only after particular components of antioxidant or epithelial reserve have been exceeded. This possibility suggests that a modifier may appear ineffective when males and females are pooled or may be protective in one sex while neutral in the other.

Dietary intervention studies further demonstrate both the strengths and the interpretive boundaries of the model. Two days of manganese supplementation before irradiation improved male survival at selected doses but did not produce a comparable benefit in females. The effect was associated with changes in nonenzymatic manganese speciation rather than increased MnSOD2 abundance. This identifies manganese-dependent redox chemistry as a candidate mechanism, but the study’s primary endpoint was survival. It therefore establishes an organismal radiation modifier more directly than a gastrointestinal countermeasure [37].

In contrast, prefeeding with *A. pullulans* connected an orally delivered intervention to both survival and intestinal morphology. The treatment improved male post-irradiation lifespan and reduced abnormal enterocyte nuclear morphology. However, protection was species-specific: *Rhodotorula taiwanensis* did not confer the same benefit and could reduce survival under some conditions. Moreover, improvement in nuclear morphology did not completely restore actin-associated epithelial organization. These findings reinforce the need to test candidate countermeasures across multiple endpoints rather than assuming that radioresistance of the dietary organism, antioxidant activity, or improvement in one morphological feature will necessarily translate into comprehensive tissue protection [38].

Microbial status provides a similar lesson. Conventional microbes appeared protective against some delayed oxidative, mitochondrial, and survival effects following developmental irradiation, yet radiation also altered microbial abundance and reduced diversity [35]. Defined *L. reuteri* feeding further showed that a particular microbial intervention can improve survival together with several gut-associated endpoints, although combined pre- and post-exposure dosing prevented clean classification as radioprotection or mitigation [55]. The microbiome should therefore not be treated as a binary variable in which the presence of microbes is either beneficial or harmful. Defined communities, controlled diets, separated dosing windows, and longitudinal analysis will be needed to determine which organisms or metabolites preserve epithelial function and which amplify injury after barrier disruption.

Survival remains an important endpoint because it integrates the total physiological cost of exposure. However, survival alone cannot establish that an intervention acts through the intestine. Irradiation can affect neural, muscular, reproductive, metabolic, and other tissues, particularly at the high doses used in adult fly experiments. A strong claim of gastrointestinal protection therefore requires survival to be paired with at least one gut-specific functional or mechanistic endpoint, such as permeability, epithelial morphology, ISC activity, lineage behavior, microbial translocation, or intestine-restricted genetic manipulation. The value of the fly lies precisely in the ability to make these pairings within the same experimental system.

Collectively, direct irradiation studies establish that the adult *Drosophila* midgut can express multiple components of gastrointestinal radiation injury. Ionizing radiation produces DNA damage, apoptosis, altered stem cell proliferation, abnormal differentiation, differentiated-cell plasticity, morphological disruption, barrier failure, oxidative and autophagy-associated responses, immune activation, microbiome changes, and reduced survival. These phenotypes can be modified by genetic background, sex, microbial status, antioxidant capacity, regenerative signaling, and dietary intervention.

The evidence also reveals a field that remains methodologically heterogeneous. Studies differ in radiation source, dose, dose rate, life stage, genotype, sex, diet, microbial state, and post-exposure interval. Some directly measure intestinal function, whereas others infer gastrointestinal involvement from survival or whole-animal physiology. The studies should not be collapsed into a single dose–response curve, but their diversity can be used productively to identify which variables control the transition from repair to persistent dysfunction.

Progress will require greater methodological integration. Cellular damage, regenerative activity, epithelial organization, barrier function, microbial behavior, feeding, and organismal outcome must be measured as related but noninterchangeable dimensions. Table 2 summarizes the principal studies, separates core intestinal injury evidence from modifier and intervention studies, and makes explicit why exposure context, endpoint selection, and attribution strength are central to interpretation.

## 5. Experimental Approaches for Measuring Gut Injury and Recovery

The strength of the fly model depends less on any single assay than on the ability to combine measurements across biological scales. Radiation resistance is not a unitary phenotype. A fly may survive despite persistent intestinal injury, a gut may retain barrier function while containing molecularly damaged cells, and increased proliferation may represent either effective repair or dysregulated growth. Experimental designs should therefore define the specific component of injury being tested and pair it with at least one complementary functional endpoint.

A useful framework is to organize measurements into four linked levels: initiating molecular injury, cellular and lineage response, tissue organization and barrier function, and organismal consequence. Countermeasure studies should additionally measure exposure to the intervention itself, because changes in taste, feeding, absorption, or nutritional composition can masquerade as altered radiation sensitivity.

### 5.1. Radiation Exposure and Reporting

Meaningful comparison begins with complete exposure reporting. Studies should specify radiation quality and source, nominal dose and independently measured absorbed dose, dose rate, exposure geometry, container and food configuration, cohort density, temperature during irradiation, fractionation schedule, and whether flies were immobilized or allowed to move. Age, developmental stage, sex, genotype, nutritional state, and microbial status should be reported with equal care. These variables can alter delivered dose, biological reserve, or both.

The high doses often used in adult flies should not be converted into human-equivalent doses. Adult Drosophila tolerate exposures that are lethal to mammals because the dose-limiting physiology of the two organisms differs substantially. In particular, flies lack the mammalian-type hematopoietic and vascular systems that contribute strongly to whole-body radiation lethality, and proliferative somatic cells are distributed differently across adult tissues. Differences in body size, tissue organization, cell turnover, and reproductive biology further preclude direct numerical dose translation. The appropriate comparison is therefore biological effect rather than numerical dose. Investigators should identify exposure ranges that produce interpretable transitions among subclinical damage, compensatory repair, barrier dysfunction, and mortality within the fly system.

Time is a second exposure dimension. Early analyses capture DNA damage, checkpoint activity, apoptosis, and stress signaling; intermediate analyses reveal regenerative activity, differentiation, and epithelial reorganization; later analyses capture persistent permeability, dysbiosis, lifespan effects, and delayed failure. Sampling at only one interval risks misclassifying a transient arrest as stem cell loss or a temporary proliferative burst as durable recovery.

### 5.2. Molecular, Cellular, and Lineage-Resolved Endpoints

DNA damage can be assessed through gamma-H2Av foci, oxidative lesions such as 8-oxo-deoxyguanosine, comet-based approaches, or transcriptional reporters. Apoptosis may be evaluated using activated caspase staining, TUNEL, or expression of pro-apoptotic genes. Reactive oxygen species reporters, antioxidant response reporters, and biochemical measurements can characterize redox state. Autophagy-associated reporters such as Atg8 add another dimension, but puncta or reporter abundance should not be equated automatically with autophagic flux; paired measurements of formation and lysosomal turnover are needed to distinguish induction from impaired clearance [49].

ISC behavior is commonly measured by Delta-positive cell abundance, phospho-histone H3, EdU incorporation, or lineage-specific reporters. These measurements should be combined with markers of DNA damage and differentiation. A high mitotic index is not sufficient evidence of repair if proliferating cells retain damage or produce abnormal progeny. Conversely, reduced mitosis may reflect reversible checkpoint activation rather than irreversible loss of regenerative capacity.

The genetic toolkit of *Drosophila* provides a distinctive advantage for causal analysis. Cell-type-specific drivers, temperature- or drug-controlled expression systems, mosaic analysis, lineage tracing, RNA interference, overexpression, and mutant alleles allow candidate pathways to be manipulated in ISCs, enteroblasts, enterocytes, enteroendocrine cells, visceral muscle, or systemic tissues. These tools can distinguish epithelial-autonomous protection from indirect effects and determine whether an intervention acts before exposure, during damage recognition, or during regenerative recovery.

### 5.3. Morphology and Regional Tissue Analysis

Whole-gut length and gross morphology provide rapid indicators of tissue deterioration, but regional analysis is essential because the midgut is functionally compartmentalized [43,44]. Studies should define the anatomical region examined and avoid treating a posterior-midgut field as representative of the entire organ. Blinded image acquisition and predefined scoring criteria improve reproducibility, particularly for phenotypes such as abnormal nuclear shape, cell-boundary loss, epithelial holes, or dysplasia.

Confocal imaging of nuclear markers, cortical actin, junctional proteins, cell-type reporters, and basement-membrane or visceral-muscle features can connect molecular injury to tissue organization. Nuclear morphology, actin-defined boundaries, cell density, and tissue continuity should be quantified separately. Their partial independence in fungal-feeding experiments demonstrates why a single morphological score can conceal biologically important divergence among endpoints [38].

Longitudinal imaging is challenging in dissected adult guts, but cohorts sampled across multiple intervals can reconstruct the sequence of injury and recovery. Where feasible, lineage tracing should be incorporated to determine whether regenerated cells derive from surviving ISCs, altered progenitors, or plasticity within differentiated populations.

### 5.4. Barrier Function, Immune Activation, and Microbiome Endpoints

The Smurf assay provides an accessible whole-animal measure of intestinal barrier failure. Flies consume a non-absorbed blue dye, and loss of epithelial containment produces visible coloration outside the digestive tract. Because the assay depends on dye ingestion, flies that do not feed can be mistaken for animals with intact barrier function unless consumption is verified. Investigators should therefore report consumption conditions and distinguish flies that did not feed from those that retained the dye within the gut. The assay can be scored categorically or quantified by imaging or dye extraction [51]. Its simplicity supports large cohorts and genetic screens, but it measures a relatively advanced failure state that can also be influenced by gut transit, dye concentration, and scoring criteria.

Fluorescent dextran or related permeability approaches can provide quantitative alternatives, although molecular size, administration, recovery, and normalization require validation. Junctional imaging supplies structural context, but junction-protein localization is not itself proof of barrier function. The strongest designs combine direct permeability with morphology and survival.

Barrier disruption should be linked to host–microbe consequences where possible. Antimicrobial peptide expression, Imd/Relish and Toll/Dif reporters, bacterial culture or quantitative PCR from normally sterile tissues, and measurements of systemic immune activation can test whether leakage produces biological exposure beyond the lumen. Microbiome composition can be examined by culture, targeted sequencing, 16S profiling, or metagenomic approaches. Because fly microbial communities are highly sensitive to food, housing, transfer frequency, and laboratory environment, microbial methods must include appropriate cage, food, and batch controls.

Axenic and gnotobiotic animals provide the clearest causal tests. Reassociation with defined organisms can distinguish protection by microbial presence from protection by a specific species or metabolite. These experiments should be accompanied by microbial-load measurements, because a nominally defined community may change substantially after irradiation or dietary intervention [35,36].

### 5.5. Feeding, Survival, and Integrated Study Design

Survival and lifespan integrate the total physiological cost of exposure and remain important endpoints. They do not, however, establish that an intervention acts through the intestine. Whole-body irradiation can affect neural, muscular, reproductive, metabolic, and renal-like functions, particularly at high doses. A claim of gastrointestinal protection therefore requires survival to be paired with a gut-specific functional or mechanistic endpoint.

Oral delivery is a major practical advantage of the model, but it introduces a measurement obligation. Test compounds can be incorporated into solid or liquid diets, and capillary feeding assays can quantify consumption with high temporal resolution [56]. Dye-based ingestion controls, measurements of body burden, or compound-specific analytical measurements should be used when feasible. Pair-fed or calorically matched controls may be necessary when an intervention alters palatability, feeding frequency, or nutrient composition.

The most informative designs integrate endpoints across biological scales and time points: at least one molecular or cellular endpoint, one tissue or barrier endpoint, and one organismal endpoint should be measured at early, intermediate, and late intervals. Sex, age, genotype, and microbial status should be incorporated deliberately rather than treated as residual noise. Such integration allows investigators to distinguish a treatment that delays death from one that genuinely preserves epithelial function and regenerative competence.

## 6. Drosophila as a Platform for Countermeasure Discovery

*Drosophila* is most valuable in countermeasure development at the discovery and prioritization stages. The model can test many genotypes, diets, compounds, microbial states, and dosing schedules in parallel while preserving whole-animal physiology. Its role is not to establish human dosing or regulatory efficacy, but to identify mechanisms and interventions sufficiently compelling to justify validation in mammalian intestinal systems.

A productive screening strategy begins with a defined injury phenotype and proceeds through increasingly stringent filters. An initial survival or permeability screen can identify candidates; secondary assays can determine whether the effect is reproducible, sex- and genotype-dependent, dose-responsive, and associated with preserved epithelial function; cell-specific genetics can then test candidate mechanisms. Candidates that survive this sequence can be evaluated in mammalian organoids, rodent irradiation models, and, where appropriate, larger animals.

### 6.1. Genetic Discovery and Pathway Validation

Natural genetic variation and targeted manipulation allow countermeasure discovery to begin with mechanism rather than compound identity. The Drosophila Genetic Reference Panel screen performed by Sharma and colleagues illustrates this approach: strain-dependent variation in radiation-induced permeability led to identification of musashi, followed by ISC-specific validation connecting regenerative competence to barrier preservation and survival [45]. Similar screens could use permeability, epithelial morphology, lineage behavior, or delayed survival as entry phenotypes.

The meltrin/ADAM19 study demonstrates a second, more explicitly translational version of this workflow. A DGRP permeability screen nominated meltrin; enterocyte-specific knockdown reduced post-irradiation permeability and selected damage- and senescence-associated readouts; and pharmacologic inhibition of the mammalian ortholog ADAM19 with batimastat reduced permeability and inflammatory readouts in a doxorubicin-treated mouse model. ADAM19 suppression or inhibition also modified senescence-associated secretory phenotypes in human fibroblasts [54].

The mammalian experiments modeled genotoxic injury rather than radiation GI-ARS, and batimastat is a broad metalloprotease inhibitor rather than an ADAM19-specific agent. Even so, the sequence from fly screen to intestine-restricted validation to mammalian testing exemplifies how *Drosophila* can nominate a conserved target without requiring the model to establish clinical efficacy.

Cell-specific ATM/ATR and enterocyte DNA-damage-response experiments likewise show how the fly toolkit can distinguish stem cell-intrinsic repair requirements from non-autonomous responses to damaged differentiated cells [52,53]. Genetic results can nominate druggable nodes, biomarkers, and response signatures. They can also reveal when a candidate treatment acts through the intestine or through another tissue. If an intervention loses efficacy after intestine-specific reduction of a pathway component, gastrointestinal causality becomes more plausible. If efficacy persists despite intestinal pathway blockade, systemic or behavioral mechanisms should be considered.

### 6.2. Pharmacologic and Dietary Interventions

Both larvae and adults readily consume test fractions compounded into food, and adults can be maintained on liquid diets. This supports prophylactic, concurrent, and post-exposure dosing schedules without injection or repeated handling. The approach is especially well suited to compounds intended for oral administration or to dietary modifiers that may act directly within the lumen or epithelium.

Dietary manganese provides an example of an organismal modifier discovered through the fly system. Brief prophylactic feeding improved survival in irradiated males without increasing MnSOD2 protein abundance, directing attention toward nonenzymatic manganese-containing antioxidant chemistry [37]. Because the primary endpoint was survival, the result identifies a candidate radioprotective mechanism more directly than a gastrointestinal countermeasure. The next step is to connect protection to permeability, stem cell fate, epithelial redox state, and intestine-restricted genetic dependencies.

Screening should distinguish radioprotectors administered before exposure from mitigators administered after exposure. Candidates should also be tested for toxicity in nonirradiated animals, effects on feeding and development, and persistence of benefit after treatment withdrawal. A compound that merely suppresses food intake or slows proliferation may appear protective at an early endpoint while impairing long-term recovery.

### 6.3. Microbial and Fungal Interventions

The fly gut provides unusual leverage for testing microbial countermeasures because communities can be simplified and reconstructed. Conventional, antibiotic-treated, axenic, and gnotobiotic cohorts can distinguish whether benefit arises from microbial biomass, a particular organism, a metabolite, or an interaction with diet. The observation that conventional microbes reduced some delayed oxidative and survival effects after developmental irradiation shows that microbial depletion is not inherently protective [35].

Zhang and colleagues provide a proof of principle for a defined probiotic intervention. *L. reuteri* administered before and after irradiation improved survival and modified permeability, oxidative stress, ISC abundance, microbiome features, and AMPK/mTOR- and autophagy-associated readouts [55]. Because dosing spanned both sides of exposure, the experiment cannot distinguish a true pre-exposure radioprotector from a post-exposure mitigator, and the responsible bacterial product or host pathway remains uncertain. These limitations define the next screening steps: separate timing windows, quantify intake and microbial abundance, test strains and cell-free products independently, and use intestine-restricted genetics to establish mechanism.

Fungi represent a particularly interesting intersection of diet, natural history, and radiation biology. *Drosophila* are naturally associated with yeasts, and diverse fungi display substantial radiation tolerance. The recovery of fungi from the damaged Chernobyl reactor environment helped motivate broader interest in fungal radiation biology and radioprotective chemistry [57]. Radioresistant fungi can therefore be tested as whole-food interventions, sources of pigments and antioxidants, or bioprospecting targets for protective metabolites.

Direct comparison of *Aureobasidium pullulans* and *Rhodotorula taiwanensis* illustrates why candidates must be evaluated empirically rather than selected solely by their own radioresistance. *A. pullulans* improved male survival and reduced abnormal enterocyte nuclear morphology, whereas *R. taiwanensis* did not reproduce the benefit and could worsen survival under some conditions [38]. Protection was also incomplete across intestinal endpoints. These results argue for species-specific, mechanism-oriented screening rather than a general assumption that consuming a radioresistant organism will confer host radioprotection.

### 6.4. Criteria for Translational Prioritization

Candidate interventions should meet several criteria before advancement from *Drosophila* screening to mammalian validation (Table 3).

For microbial and dietary candidates, advancement should also require separation of pre-exposure protection from post-exposure mitigation and evidence that benefit is not explained by altered food intake, microbial load, or nutritional composition. Failure in one category is informative. A treatment that protects only one endpoint may still reveal a useful mechanism even if it is not a complete countermeasure.

This staged approach also aligns with the 3R framework by using *Drosophila* for early mechanistic screening and candidate prioritization, potentially reducing the number of vertebrate animals required for downstream studies and refining the selection of interventions advanced to mammalian models [58]. Vertebrate systems remain necessary for translational validation of mammalian anatomy, physiology, systemic injury, and clinical endpoints.

Translation should be organized as a funnel rather than a leap. Fly studies can identify the conserved node, optimal timing, and discriminating biomarkers. Human or murine intestinal organoids can then test epithelial-autonomous effects in mammalian tissue. Rodent models can evaluate crypt regeneration, fluid balance, microbial translocation, hematopoietic interactions, and medical management. Large-animal studies remain necessary for regulatory and clinical development. Used in this sequence, *Drosophila* can reduce the number of poorly supported candidates entering expensive vertebrate studies [2,4,5,6,7,8,29].

## 7. Limitations and Translational Boundaries

The limitations of the fly model are not incidental caveats; they define the questions the model can answer responsibly. *Drosophila* is strongest for conserved epithelial injury-response logic, genetic modifier discovery, and screening. It is weakest when the research question depends on mammalian anatomy, adaptive immunity, vascular physiology, hematopoietic failure, complex fluid management, or direct dose equivalence [59].

### 7.1. Anatomical and Physiological Divergence

The fly midgut lacks crypt–villus architecture, Paneth cells, goblet-cell mucus biology, mesenteric vasculature, and the full stromal and immune-cell environment of the mammalian intestine. Septate junctions and the peritrophic matrix preserve barrier function through structures that are functionally analogous but not homologous in every mechanistic detail to mammalian tight junctions and mucus. Findings involving barrier failure should therefore be translated at the level of physiological outcome and conserved signaling, not assumed structural identity [7,8,43,44,50].

Clinical GI-ARS develops in the context of concurrent hematopoietic, vascular, immune, neuroendocrine, renal, and metabolic injury. *Drosophila* has an open circulatory system, lacks adaptive immunity, and handles excretion and osmotic balance through insect-specific organs. It cannot reproduce medical management, sepsis, fluid replacement, or multi-organ syndromic interactions with human fidelity. These components must be evaluated in vertebrate models [2,4,5,6,7,8].

### 7.2. Dose, Radiation Quality, and Dosimetry

The extraordinary radiation tolerance of adult flies creates both opportunity and risk. High exposures generate a broad phenotypic range, but they may also produce mechanisms that are not dominant at clinically relevant mammalian doses. Numerical dose translation is inappropriate. Investigators should instead report absorbed dose rigorously, define the biological effect range within the fly, and test whether mechanisms persist across lower and higher injury severities [59].

Radiation quality and dose rate may matter as much as total dose. Most intestinal studies have used acute photon exposures. Proton, neutron, heavy-ion, mixed-field, chronic low-dose-rate, and fractionated exposures remain comparatively underexplored. Container geometry, movement of flies during irradiation, attenuation by food, and dose gradients within vials can all introduce variability. Dosimetry should be matched to the actual experimental configuration rather than inferred from machine output alone [59].

### 7.3. Biological Context and Reproducibility

Age, developmental stage, sex, genetic background, diet, circadian timing, cohort density, and microbiome state can modify radiation phenotypes. Developmental irradiation may produce adult outcomes through altered organ formation rather than injury to a mature epithelium. Male and female effects may diverge at selected doses or under particular dietary conditions. A phenotype observed in one laboratory stock should not be assumed to generalize across the species [35,37,38,45,46,54,55].

Furthermore, the microbiome is especially sensitive to husbandry. Food formulation, preservatives, transfer schedule, environmental microbes, and co-housing can reshape community composition. Dietary interventions may alter both the host and the microbes, making mechanism difficult to assign without axenic or defined-community controls. Reproducibility will require explicit reporting of food preparation, microbial methods, and environmental conditions [35,36,55].

### 7.4. Endpoint Attribution and Translational Claims

Whole-animal survival is powerful but nonspecific. A candidate can extend lifespan through neural, muscular, reproductive, behavioral, or metabolic effects without preserving the gut. Conversely, an intestine-specific benefit may not improve survival if another organ remains dose-limiting. Strong gastrointestinal claims require direct intestinal endpoints and, where possible, intestine-restricted manipulation [37,38,45,54].

The direct intestinal radiation literature remains small, and several conclusions depend on single studies. Negative results are particularly difficult to interpret when feeding, delivered dose, or assay sensitivity are uncertain. The field should resist premature claims that a pathway or intervention is universally protective. The proper translational statement is narrower: the fly can identify conserved mechanisms and prioritize candidates, while mammalian systems determine whether those findings operate within clinical GI-ARS [35,37,38,45,46,47,48,49,52,53,54,55,59].

## 8. Future Directions

Future progress will depend not only on additional studies, but also on clearer reporting and greater standardization across experimental designs. A community reporting framework should specify the minimum information to be reported for radiation source, quality, dose, dose rate, geometry, dosimetry, fly age, sex, genotype, density, diet, microbial status, and analysis interval. One or more standardized reference exposure protocols could provide common points of comparison while preserving the flexibility to study other doses and radiation qualities [59].

Future experiments should be longitudinal and multi-endpoint. Early DNA damage and stress signaling should be connected to later lineage behavior, epithelial organization, permeability, microbiome state, and survival. The same candidate should be evaluated across these layers so that partial protection and mechanistic tradeoffs become visible. Standardized permeability and morphology scoring, blinded analysis, and preregistered exclusion criteria would strengthen reproducibility [38,45,54,55].

Cell-resolved approaches can clarify which populations initiate and sustain injury. Single-cell and spatial transcriptomics, lineage tracing, mosaic genetics, proteomics, metabolomics, and improved live or ex vivo imaging can distinguish damaged ISCs from stressed enterocytes, plastic enteroendocrine cells, and altered niche tissues. These approaches are particularly suited to determining whether repair restores the pre-injury epithelium or creates a persistent regenerative state with altered function [43,44,48,52,53].

Microbiome studies should move from presence-versus-absence comparisons toward defined ecological and metabolic hypotheses. Gnotobiotic communities can test whether selected bacteria or fungi alter redox balance, epithelial signaling, nutrient availability, or barrier repair. Longitudinal measurements are needed because a community that is protective before irradiation may become harmful after barrier failure, and the same taxon may exert different effects under different diets [35,36,55].

Sex, age, and genetic diversity should be incorporated at the discovery stage. The contrasting sex effects observed across studies indicate that pooling can conceal meaningful biology. Genetic panels and selected wild-derived backgrounds can identify response modules that are robust across diversity and those that depend on specific host states. This information can improve prioritization before mammalian validation [37,38,45,54].

The radiation landscape should also broaden. Fractionated exposure, low-dose-rate irradiation, proton and heavy-ion radiation, and combined stressors relevant to spaceflight or radiotherapy may reveal mechanisms not visible after acute gamma exposure. Such studies should remain anchored to clear gastrointestinal endpoints rather than relying on organismal survival alone [59].

Finally, the fly should be integrated deliberately with mammalian models. Parallel perturbation of a conserved pathway in flies and intestinal organoids can test epithelial autonomy; rodent studies can add crypt architecture, adaptive immunity, microbiome complexity, and systemic injury; larger animals can address clinical management and regulatory endpoints. The most valuable future is not one in which *Drosophila* competes with these systems, but one in which each model performs the part of the translational sequence for which it is best suited [2,4,5,6,7,8,29].

## 9. Conclusions

*Drosophila melanogaster* has moved from a foundational model of radiation-induced mutation to a tractable system for studying how a living gastrointestinal epithelium responds to injury. Evidence from the adult midgut shows that irradiation can disrupt epithelial renewal, differentiation, barrier integrity, immune signaling, host–microbe interactions, and organismal survival. These responses are not fixed, but vary with genotype, sex, diet, microbial state, redox capacity, and regenerative signaling, underscoring the value of the model for identifying the biological factors that shift the intestine toward recovery or failure. The model’s principal contribution is resolution. It can separate DNA damage from cell death, proliferation from successful regeneration, morphology from barrier function, and organismal survival from intestine-specific protection. Its genetic and experimental scale makes it especially valuable for discovering causal mechanisms and narrowing large candidate spaces before vertebrate validation.

The fly midgut does not replicate the mammalian intestine in miniature. It provides a compact and powerful system for determining how an epithelium senses radiation damage, decides which cells will survive, restores or loses tissue organization, interacts with microbes, and ultimately recovers or fails. That role positions *Drosophila* as a complementary bridge between reductionist cellular models and translational mammalian radiobiology. Its greatest value will come from using that bridge deliberately: to identify conserved mechanisms, prioritize candidate countermeasures, and advance only the strongest findings into mammalian validation.

## Figures and Tables

**Table 1:** Conserved or functionally analogous epithelial injury-response modules linking the Drosophila midgut to mammalian gastrointestinal radiation biology.

Injury-Response Module	Drosophila Pathway/Readouts	Mammalian Analog	Relevance to GI Radiation Injury	References
Cytokine-driven epithelial repair	Upd2/Upd3-Domeless-Hopscotch-Stat92E; pSTAT/Stat92E reporters, upd3, Socs36E, mitotic ISC counts	IL-6-family cytokines/gp130-JAK/STAT3; pSTAT3, SOCS3, IL-6/IL-11	Converts epithelial and microbial stress into regenerative signaling; helps determine whether the response resolves injury or amplifies inflammation.	[9,10,11,12]
Stress-activated MAPK signaling	JNK/Basket/AP-1 and p38-like responses; puc-lacZ, pJNK, ROS assays, AP-1 targets	JNK/p38/AP-1 stress signaling	Senses ROS, DNA damage, infection, and dying cells; may drive apoptosis, inflammatory signaling, compensatory proliferation, or repair according to timing and intensity.	[13,14,15,16]
EGFR/Ras/ERK repair competence	Vein, Spitz, and Keren; dpERK, ISC proliferation, epithelial renewal assays	EGF-family ligands/EGFR/Ras/ERK; pERK, Ki-67, wound-repair readouts	Supports epithelial restitution, proliferative competence of surviving progenitors, and regenerative recovery after injury.	[16,17,18,19]
Wnt/Wingless stem cell maintenance	Wingless/Armadillo; Wg targets, ISC maintenance, regional patterning	Wnt/beta-catenin; LGR5, AXIN2, OLFM4, crypt regeneration	Maintains regenerative epithelial compartments vulnerable to radiation and influences stem cell survival and repair capacity.	[20,21,22,23,24]
Notch/Delta lineage allocation	Delta-Notch; Notch reporters, enterocyte versus enteroendocrine balance	DLL/Jagged-Notch-HES/HEY; absorptive versus secretory lineage markers	Coordinates differentiation after injury so that epithelial replacement restores appropriate cell types rather than disordered repair.	[25,26,27,28,29]
Hippo/Yorkie growth and plasticity control	Hippo/Yorkie; nuclear Yki, diap1, expanded, ISC proliferation	Hippo/YAP/TAZ; nuclear YAP/TAZ, CTGF, CYR61	Links epithelial stress to growth, plasticity, and wound-repair states; excessive activation may promote hyperplasia or dysplasia.	[30,31,32,33,34]
Innate immune and microbial sensing	Imd/Relish and Toll/Dif; Diptericin, Drosomycin, immune reporters	TLR/NOD/TNF/IL-1-IKK-NF-kappaB; cytokines and antimicrobial peptides	Connects dysbiosis and microbial products to epithelial inflammation, antimicrobial defense, barrier injury, and systemic decline.	[35,45]
Barrier and junctional integrity	Smooth septate junctions; Mesh, Ssk, Coracle, Dlg; Smurf and permeability assays	Tight and adherens junction networks; claudins, occludin, ZO-1, E-cadherin; TEER and dextran permeability	Provides a functional readout of epithelial failure and risk of microbial translocation, dehydration, inflammatory amplification, and mortality.	[45,50,51]
Oxidative-stress defense	Keap1/CncC; gstD-GFP, antioxidant gene induction, ROS assays	KEAP1/NRF2; HO-1, NQO1, GCLC/GCLM, glutathione pathways	Controls redox resilience after radiation-induced ROS and may mediate diet-, microbiome-, fungal-, or drug-associated protection.	[14,35,37,38]
DNA damage, apoptosis, and repair fate	p53, gamma-H2Av, caspases, TUNEL, mitotic arrest, ISC proliferation	p53, gamma-H2AX, p21, caspase-3, DNA-repair checkpoints	Determines whether irradiated cells arrest, repair, die, or re-enter regenerative programs needed for tissue recovery.	[45,46,47,48,49]

Abbreviations: GI, gastrointestinal; ISC, intestinal stem cell; ROS, reactive oxygen species; TEER, transepithelial electrical resistance. Relationships indicate conserved or analogous injury-response logic, not anatomical equivalence.

**Table 2:** Evidence matrix of Drosophila irradiation studies relevant to intestinal injury mechanisms, biological modifiers, and countermeasure discovery.

Study	Life Stage/Biological Context	Radiation Source/Quality	Dose/Schedule	Dose Rate	Analysis Interval	Gut-Relevant Endpoints	Modifier or Intervention	Principal Finding	Interpretive Limitation
**Core intestinal injury and mechanism studies**									
Pyo et al. [46]	Seven-day-old adults; ISC-focused analysis.	Gamma rays	2 or 10 Gy; acute whole-body exposure	2.25 Gy/min	Acute post-exposure; assay-specific.	gamma-H2Av, 8-oxo-dG, ISC mitosis, JNK/AKT signaling, differentiation, centrosome number.	Dose comparison.	2 Gy increased ISC proliferation despite persistent damage, abnormal differentiation, and centrosome amplification; 10 Gy reduced proliferative capacity.	Cellular and lineage study without direct permeability or survival endpoints.
Xing et al. [47]	Young adults; posterior midgut plus germline stem cell model.	Ionizing radiation	50 Gy; acute whole-body exposure	NR	Early apoptosis through approximately 6 days.	Differentiated-cell versus esg-positive progenitor apoptosis; post-irradiation ISC lineage output.	Cell-type susceptibility; Pvf1-Tie-bantam-Hid survival axis.	ISCs and enteroblasts were less apoptosis-prone than differentiated cells and retained multilineage output, although post-irradiation clones were smaller.	Detailed causal analysis of the survival axis was performed mainly in the germline; no barrier endpoint.
Park et al. [52]	Adult flies; ISC/enteroblast-specific ATM or ATR RNAi.	Cesium-137 gamma rays	5 Gy	2.55 Gy/min	1 h after irradiation; longer genetic homeostasis assays.	pS/TQ and gamma-H2AvD in progenitors; ISC abundance and EdU incorporation in complementary assays.	ATM and/or ATR depletion in ISCs and enteroblasts.	Irradiation activated progenitor DNA-damage responses, with stronger dependence on ATR; ATM/ATR depletion impaired ISC maintenance and proliferation.	Radiation served mainly as an acute DNA-damage challenge; longer phenotypes were not longitudinal post-irradiation recovery measures.
Park et al. [53]	Adult flies; enterocyte-specific depletion of DNA-damage-response genes.	Cesium-137 gamma rays	5 Gy	2.55 Gy/min	1 h after irradiation; chronic knockdown assays over days.	gamma-H2AvD in enterocytes and progenitors; enterocyte death, ISC proliferation, and gut-aging phenotypes in complementary assays.	Enterocyte-specific Mre11, Rad50, Nbs1, ATM, ATR, Chk1, or Chk2 RNAi.	Acute irradiation activated DNA-damage responses in enterocytes and progenitors; chronic enterocyte DDR deficiency promoted enterocyte loss and non-autonomous ISC hyperproliferation.	The major tissue-aging phenotypes arose from chronic genetic DDR deficiency, not a longitudinal irradiated-recovery experiment.
Sharma et al. [45]	Five-day-old adults of both sexes; 156 DGRP lines and ISC-specific manipulations.	320-kV X-rays	100 Gy acute or 4 x 25 Gy every other day	NR	30 min to 14 days.	gamma-H2Av, apoptosis, hid/reaper/upd3 and antimicrobial genes, pH3 and Delta-positive ISCs, gut length, Smurf permeability, survival.	Natural variation; ISC-specific musashi loss or gain; Cyclin E.	Radiation caused persistent loss of regenerative capacity and late barrier failure; musashi or forced ISC proliferation reduced permeability and modestly improved survival.	High-dose whole-body model; survival is nonspecific, although intestine-restricted genetics strengthen gut attribution.
Trinca and Malik [49]	Three- to six-day-old adults; mCherry-Atg8 reporter in enterocytes.	Cesium-137 gamma rays	150 Gy	0.43 Gy/min	1 day.	mCherry-Atg8 abundance and puncta in posterior-midgut enterocytes.	None.	Irradiation increased an autophagy-associated reporter throughout much of the midgut.	Reporter abundance and puncta do not establish autophagic flux, functional direction, or barrier preservation.
Qian et al. [48]	Adult enteroendocrine lineage; lineage tracing and single-cell transcriptomics.	X-rays	50 or 100 Gy; acute whole-body exposure	NR	Hours after exposure through lineage-tracing endpoints.	Enteroendocrine lineage conversion; Prospero, Delta, progenitor genes, Xrp1, upd3, and transcriptional state.	Enteroendocrine-specific Xrp1 or upd3 loss; Xrp1 overexpression.	Radiation induced enteroendocrine-cell plasticity; Xrp1 and upd3 were required, and Xrp1 was sufficient to induce progenitor-associated gene expression.	No barrier or survival endpoint; durable functional contribution of converted cells remains unresolved.
**Biological modifiers and candidate interventions**									
Lee et al. [35]	Third-instar larvae followed into adulthood; conventional and axenic cohorts.	Cesium-137 gamma rays	0.1 or 5 Gy	0.67 cGy/min (0.1 Gy); 3.25 Gy/min (5 Gy)	Development through adult life; selected assays near 14 days.	Microbial abundance and diversity, intestinal ROS, mitochondrial physiology, lifespan, locomotion, and reproduction.	Conventional versus axenic microbial state.	Irradiation altered microbial abundance and diversity; axenic flies showed greater delayed oxidative and mitochondrial responses and dose-dependent lifespan loss.	Developmental exposure confounds mature-gut injury with altered development; several outcomes are organismal rather than gut-specific.
Zhang et al. [55]	Adult females; probiotic feeding.	Gamma rays; source NR	100 Gy	NR	Six days of prefeeding, continued after exposure; follow-up to approximately 14 days.	Survival, Smurf permeability, intestinal ROS and oxidative markers, ISC abundance, microbiota, and AMPK/mTOR/autophagy-associated readouts.	*Lactobacillus reuteri* DSM 17938 and/or ATCC PTA-6475, 1 x 10^9 CFU/mL, before and after irradiation.	*L. reuteri* improved survival and attenuated several intestinal oxidative, permeability, and regenerative abnormalities; responses varied by preparation.	Combined prophylactic and post-exposure dosing prevents clean protector-versus-mitigator assignment; intestine-specific causality was not established.
Bar et al. [54]	Five-day-old adult females; enterocyte-specific meltrin RNAi.	320-kV, 10-mA X-rays	100 Gy over 10 min	NR	5 to 14 days, depending on endpoint.	Smurf permeability, gamma-H2Av, beta-galactosidase, upd3, apoptosis-associated staining, and lifespan.	Enterocyte-specific meltrin RNAi; 50 micromolar batimastat (BB-94).	Meltrin knockdown reduced post-irradiation permeability, DNA-damage and senescence-associated markers; BB-94 also reduced permeability.	BB-94 is broad-spectrum; the marker panel does not alone prove canonical mammalian senescence; fly lifespan was not improved.
Volpe et al. [37]	Newly eclosed adult males and females; liquid-diet metal prefeeding.	Vendor-calibrated cesium-137 gamma rays	700 or 1000 Gy	Approx. 12.35 Gy/min	Longitudinal survival and whole-adult biochemical analysis; MnSOD2 at 24 h.	No direct gut-specific endpoint; survival, sex effects, MnSOD2 abundance, and whole-animal manganese speciation.	5 or 10 micromolar MnCl2 for 2 days before irradiation; copper and nickel controls.	MnCl2 improved male survival at selected doses without increasing MnSOD2; spectroscopy implicated nonenzymatic high-symmetry manganese-metabolite complexes.	Establishes organismal radioprotection, not gastrointestinal protection; survival and biochemical readouts are whole-animal.
Volpe et al. [38]	Newly eclosed adult males and females; fungal prefeeding; R4 midgut analysis.	Vendor-calibrated cesium-137 gamma rays	700 or 1000 Gy	Approx. 12.62 Gy/min	2 days for gut morphology; longitudinal survival.	Enterocyte nuclear morphology, actin-defined cellular boundaries and epithelial holes, and sex-specific survival.	Two-day prefeeding with *Aureobasidium pullulans* or *Rhodotorula taiwanensis*, irradiated or nonirradiated.	*A. pullulans* improved male survival and nuclear morphology but did not fully preserve actin architecture; *R. taiwanensis* was not protective and could be detrimental.	No direct permeability assay; high whole-body dose; effects were endpoint-specific and the protective mechanism remains unresolved.

NR, not reported. Studies are grouped by primary experimental role. Inclusion does not imply equivalent strength of gastrointestinal attribution. Exposure variables are separated to highlight cross-study heterogeneity, not to support direct cross-study or cross-species dose equivalence. Full source, geometry, dosimetry, husbandry, and timing should be consulted in the original reports.

**Table 3:** Criteria for translational prioritization of candidate gastrointestinal radiation countermeasures identified in *Drosophila*.

Criterion	Recommended Evidence
Reproducibility	Benefit reproduced across independent experimental cohorts.
Efficacy across exposures	Protective or mitigating activity demonstrated at more than one biologically informative radiation exposure.
Gut-specific functional benefit	Preservation of at least one gastrointestinal endpoint, such as barrier function, epithelial morphology, regenerative activity, or lineage behavior.
Toxicity and feeding profile	Acceptable effects in nonirradiated animals, with benefit not attributable to altered feeding, palatability, or nutritional intake.
Mechanistic evidence	Evidence for a defined pathway, cellular mechanism, or response signature associated with protection or recovery.
Biological robustness	Activity demonstrated, where feasible, across more than one sex, genetic background, or relevant biological state.

## Data Availability

No new data were created or analyzed in this review. Data sharing is not applicable to this article.
